# Predictors of Minimum Acceptable Diet among Children Aged 6–23 Months in Nepal: A Multilevel Analysis of Nepal Multiple Indicator Cluster Survey 2019

**DOI:** 10.3390/nu14173669

**Published:** 2022-09-05

**Authors:** Suman Sapkota, Bipin Thapa, Amrita Gyawali, Yifei Hu

**Affiliations:** 1 Department of Child and Adolescent Health and Maternal Care, School of Public Health, Capital Medical University, Beijing 100069, China; 2 Ministry of Health and Population, Ramshahpath, Kathmandu 44600, Nepal

**Keywords:** minimum acceptable diet (MAD), infant and young child feeding (IYCF), nutrition, Nepal, children under 2 years of age

## Abstract

Background: Minimum Acceptable Diet (MAD), developed by the WHO and UNICEF, is a binary indicator of infant and young child feeding practice that assesses the quality and sufficiency of a child’s diet between the ages of 6 and 23 months. Identifying factors associated with MAD among children can inform policymakers to improve children’s nutritional status. Methods: We extracted data of 1930 children aged 6–23 months from the Nepal Multiple Indicator Cluster Survey 2019. Multilevel analysis was performed to identify factors associated with MAD. Results: Only 30.1% of the children received MAD. Children aged 13–18 months [aOR (Adjusted odds ratio): 2.37, 95% CI (95% Confidence Interval): 1.77, 3.17] and 19–23 months (aOR: 2.6, 95% CI: 1.95, 3.47) were more likely to receive MAD than children aged 6–12 months. Early breastfed children (aOR: 1.34, 95% CI: 1.05, 1.72), those currently breastfeeding (aOR: 4.13, 95% CI: 2.21, 7.69) and children without siblings aged under five (aOR: 1.33, 95% CI: 1.03, 1.73) were more likely to receive MAD. Younger maternal age (aOR: 0.97, 95% CI: 0.95–1.0), higher level of mother’s education (aOR: 1.04, 95% CI: 1.0–1.08) and more media exposure among mothers (aOR: 1.66, 95% CI: 1.24, 2.21) were positive predictors of MAD. Relatively disadvantaged ethnicity/caste (aOR: 0.71, 95% CI: 0.53, 0.94), rural residence (aOR: 1.45, 95% CI: 1.06, 2.00) and residing in Madhesh province (aOR: 0.61, 95% CI: 0.37, 1.0) were also significant predictors of MAD. Conclusions: Children aged 6–12 months, without appropriate breastfeeding, having under-five years siblings, with older mother or mother without media exposure or low education, from relatively disadvantaged ethnicity/caste, from urban areas and residing in Madhesh Province were less likely to receive MAD. Our findings can inform infant and young child feeding policies and practices in Nepal.

## 1. Introduction

Undernutrition among children is a significant public health issue, especially in low- and middle-income countries (LMICs). In 2020, Asia was home to more than half of the 149.2 million stunted children and more than two-thirds of the 45.5 million wasted children under the age of five years [1]. These undernourished children generally experience detrimental consequences regarding healthy development, survival and the occurrence of acute and chronic diseases that impact individual and societal economic productivity [2].

With one of the fastest declines in childhood stunting in the world over the past three decades, Nepal has accomplished an outstanding reduction in childhood undernutrition [3]. Parallelly, infant and young child feeding (IYCF) practices have improved significantly across several important determinants including age of child, sex of child, province of residence, household wealth quintile, ecological zone, ethnicity/caste, mother’s education, household size, and household food security from 2011 to 2016 [4]. However, among children under the age of five years, the prevalence of stunting, wasting, and underweight remained alarmingly high at 31.5%, 12%, and 24.3%, respectively [5].

Minimum acceptable diet (MAD) is a composite indicator formulated from minimum dietary diversity (MDD) and minimum meal frequency (MMF), designed to measure dietary intake for breastfeeding children and MDD and MMF along with minimum milk feeding frequency (MMFF) for non-breastfeeding children [5,6]. MAD as an indicator of IYCF practices was first proposed by the WHO working group on IYCF indicators in 2006, and later finalized in the WHO Global Consensus meeting on indicators of IYCF in 2007 [7]. An inter-agency consultation by a broad group of experts convened by the WHO and UNICEF in 2017 and 2018 revised the indicators in the present form and it was published as one of the 17 indicators of IYCF practices [6]. According to the most recent estimate, the prevalence of the minimum acceptable diet (MAD) is still concerningly low at 36% in Nepal [8].

Breastmilk and complementary foods constitute the majority of an infant’s and young child’s diet. Evidence suggests that appropriate IYCF practices (including optimal breastfeeding and appropriate complementary feeding) early in life, particularly in the first 1000 days, decrease the risk of stunting, wasting and underweight in children [2,9,10]. Therefore, the status and predictors of IYCF practices among children in Nepal need to be understood contextually. Previous studies suggest that along with characteristics of children, several other maternal, household and environmental factors could influence IYCF behaviors [4,11,12,13,14,15]. However, previous studies in Nepal are either limited by their geographic coverage or have used the former definition of MAD [4,11,12,13,14,15]. This study overcomes the limitations of the previous studies by utilizing a nationally representative sample to identify the contextual predictors of MAD among children aged 6–23 months in Nepal, using the latest indicator definition of MAD devised by the WHO and UNICEF in 2021.

## 2. Materials and Methods

### 2.1. Data Source

Data from the nationally representative Nepal Multiple Indicator Cluster Survey (MICS) conducted in 2019 in Nepal were used for this study. The survey was conducted by the Central Bureau of Statistics with support from United Nations Children’s Fund. The survey adopted a two-stage, stratified cluster random sampling. Firstly, Nepal was divided into 15 strata based on seven provinces and rural or urban areas. There were 14 provincial rural and urban strata and an additional stratum for Kathmandu valley. Then 36,020 census enumeration areas were listed as primary sampling units (PSUs). At the first stage, a total of 512 PSUs were selected using systematic probability proportionate to size (PPS) method separately for each stratum. The listing of households was done for each selected PSU and systematic random sampling was used to select 25 households from each PSU in the second stage. It was ensured that each PSU included at least 13 households with children under five years of age. Then the MICS questionnaire for children under 5 was administered to mothers or caretakers of the selected children in the household through face-to-face interviews. All the variables in this study were measured and recorded based on the response of mothers or caretakers during the interviews. The detailed methodology for the survey has been documented elsewhere [5].

For the present study, we extracted data of all the children aged 6 to 23 months (183 days to 729 days) from the publicly available children dataset of the survey (https://mics.unicef.org/surveys, accessed on 5 January 2022). The information on households and mothers was obtained by merging the household and women dataset to the children dataset. A total of 1930 children from 494 PSUs were considered for analysis after excluding children from ineligible age groups (*n* = 4683), missing values for children without data on food consumption (*n* = 19), and missing values for early initiation of breastfeeding (*n* = 9). Only 494 of the 512 PSUs were included in the final analysis, leaving 18 PSUs out because they did not have any child aged 6–23 months or had missing data. The use of all the eligible data for children aged 6–23 months from the MICS dataset allowed us to perform an analysis that provides the nationally representative estimates on prevalence of MAD and its predictors in Nepal.

### 2.2. Outcome Variable

**Figure 1:**
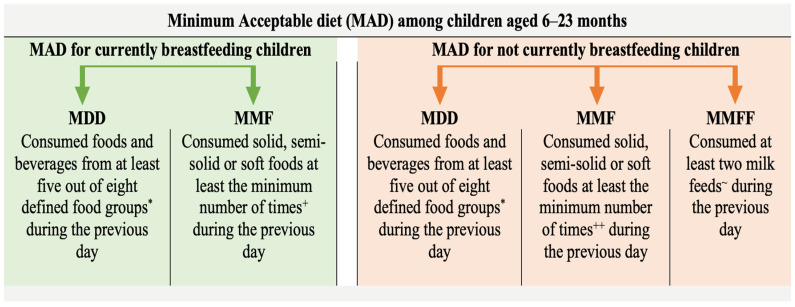
Diagrammatic representation of the definition of minimum acceptable diet. MDD: Minimum Dietary Diversity, MMF: Minimum Meal Frequency, MMFF: Minimum Milk Feeding Frequency. * Food groups: (i) Breast milk, (ii) Grains, roots, tubers and plantains, (iii) Pulses, nuts and seeds, (iv) Dairy products, (v) Flesh foods, (vi) Eggs, (vii) Vitamin-A rich fruits and vegetables, (viii) Other fruits and vegetables. ^+^ Two and three feedings of solid, semi-solid or soft foods for children aged 6 to 8 months and 9 to 23 months, respectively; ^++^ Four feedings of solid, semi-solid or soft foods or milk feeds for infants aged 6 to 23 months whereby at least one of the four feeds must be a solid, semi-solid or soft feed. ~ Formula feed or any animal milk except human milk or semi-solid or fluid yoghurt or fermented products made with animal milk.

The outcome variable for this study is MAD. Operationally, MAD for currently breastfeeding children is defined as “receiving at least the MDD and MMF for their age during the previous day.” Similarly, MAD for children not currently breastfeeding is defined as “receiving at least the MDD and MMF for their age during the previous day as well as at least two milk feeds” [6]. The diagrammatic representation of the definition of MAD is presented in Figure 1.

### 2.3. Explanatory Variables

**Figure 2:**
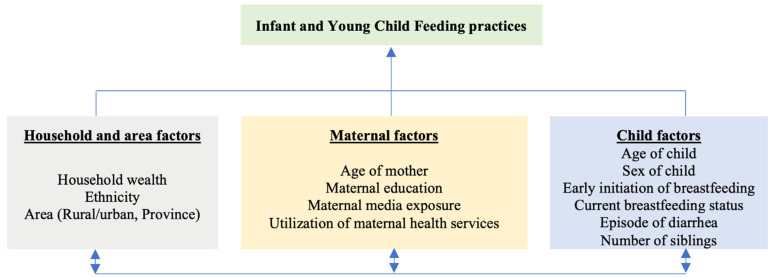
Conceptual framework of the study.

Explanatory variables for the study were selected based on the literature review and the availability of data in the MICS dataset. In addition to child and mother’s variables, contextual variables related to households and geographic areas that might affect child feeding practices were considered based on the conceptual framework of the study as illustrated in Figure 2.

Child variables include age, sex, status of early initiation of breastfeeding, current breastfeeding status, episode of diarrhea in past two weeks and status on presence of siblings aged under five in the household. Maternal variables include age, completed years of education, media exposure and use of maternal health services. The household variables were: raw household wealth score generated by principal component analysis of household possession characteristics and access to basic services [5], and ethnicity/caste of the household head. Ethnicity/caste is categorized into relatively advantaged and relatively disadvantaged groups. Relatively advantaged groups include socially and economically privileged ethnicity/caste groups which include Brahmin, Chhteri and Newar, along with relatively affluent indigenous caste groups (Janajatis) of Hilly regions of Nepal (Gurung, Magar and Thakali). Other indigenous caste groups (Janajatis) of Hilly and Terai region, socially oppressed groups (Dalits), Muslims and other ethnicity/caste groups are categorized as relatively disadvantaged [16]. Similarly, the status of urbanization and province of residence were geographical area variables. The description and measurement of explanatory variables is presented in Table 1.

### 2.4. Data Analysis

Data were analyzed using R software version 4.1.2 “Bird Hippie” (The R Foundation for Statistical Computing, Vienna, Austria) with survey and lme4 packages. These packages allow the incorporation of all the design elements of the complex survey including stratification, clustering and sampling weights in descriptive as well as multilevel multivariable analyses. The characteristics of the children, mothers, households and area along with feeding practices were computed as frequency and percentage. Bivariate logistic regression of explanatory variables with MAD was done using ‘svyglm’ function of survey package.

Owing to the hierarchical and nested structure of the data, weighted multilevel random intercept multiple logistic regression analysis was performed using the ‘glmer’ function of lme4 package. Households, along with mothers and children, were nested within PSUs and random effect of PSU was investigated. Thus, characteristics of children, mothers and households were considered as level 1 variables and area characteristics were considered as level 2 variables. No multicollinearity among explanatory variables was found (largest VIF = 2.06). Four models were formulated: Model 1 was ‘null model’ with PSU as random parameter and no fixed parameters; Model 2 consisted of child variables as fixed parameters; Model 3 consisted of child, maternal and household variables as fixed parameters; and Model 4 consisted of child, maternal, household and area variables as fixed parameters. Our statistical analyses addressed the elements of complex survey design including stratification, clustering and sampling weights. A two-sided *p*-value of less than 0.05 was considered statistically significant.

## 3. Results

The median age of children was 14 [Range (Minimum, Maximum): 6, 23] months. More than half (54.2%) of the children were male. Around two-fifths (41.5%) of the children had early initiation of breastfeeding and 93.6% of the children were currently breastfeeding. Over one-tenth (13.1%) of the children had at least one episode of diarrhea in the past two weeks. Most (62%) of the children were the single living child aged under 5 years in the household. The median age of mothers was 25 [Range (Min, Max): 15, 49] years. The median years of completed education of mothers was 8 [Range: 0, 14] years. Two-thirds (66.3%) of the mothers had utilized maternal health services during their last pregnancy and childbirth. The median household wealth score was −0.01 [Range: −1.89 to 2.42]. Almost two-thirds (63.2%) of the households were from relatively advantaged ethnicity/caste. Madhesh Province and Karnali Province accounted for 21.4% and 6.3% of the households, respectively (Table 2).

The prevalence of MAD among children was 30.1%. MDD was met among 39.8% of children, while over two-thirds (68.5%) of the children met MMF. MMFF was present among 17.8% of the non-breastfeeding children.

In the bivariate analysis, MAD was significantly associated with age of the child, current breastfeeding status, presence of siblings of age under 5 years in the household, mother’s education, media exposure, utilization of maternal health service, ethnicity/caste, combined household wealth score and province of residence. In comparison to children aged 6–12 months, children aged 13–18 months [Odds ratio (OR): 1.85, 95% confidence interval (95% CI): 1.34, 2.57] and children aged 19–23 months (OR: 2.1, 95% CI: 1.56, 2.83) were more likely to receive MAD. The children currently breastfeeding were three times (OR: 3.05, 95% CI: 1.03, 9.04) more likely to receive MAD than non-breastfeeding children. Similarly, children with no sibling aged under 5 were 1.77 times (OR: 1.77, 95% CI: 1.36, 2.30) more likely to receive MAD. With an increment in one year of completed education among mothers, there was a nine percent (OR: 1.09, 95% CI: 1.06, 1.12) increment in the odds of receiving MAD. Children whose mothers had media exposure were 2.07 times (OR: 2.07, 95% CI: 1.62, 2.65) more likely to receive MAD than their counterparts. Similarly, mothers who utilized maternal health services during their last pregnancy and delivery were 1.58 (OR: 1.58, 95% CI: 1.19, 2.09) times more likely to offer MAD for their children than those who did not. With a unit increase in household wealth score, children were 23% (OR: 1.23, 95% CI: 1.07, 1.41) more likely to receive MAD. Children from relatively disadvantaged ethnicity/caste were 45% (OR: 0.55, 95% CI: 0.42, 0.72) less likely to receive MAD compared to their counterparts. Compared with children from Province 1, those from Madhesh province and Karnali Province were 45% (OR: 0.55, 95% CI: 0.35, 0.85) and 43% (OR: 0.57, 95% CI: 0.34, 0.96) less likely to have MAD, respectively (Table 3).

In the multilevel analysis, age of child, current breastfeeding status of child and having a sibling aged under 5 in the household were predictors of MAD in Model 1. Early initiation of breastfeeding, age of mother, educational attainment of mother, mother’s media exposure and ethnicity/caste were significant predictors in Model 2. In Model 3, settlement and Province of residence were significant correlates of MAD among children along with other significant predictors of Model 2. In Model 3, compared to children aged 6–12 months, children aged 13–18 months were 2.37 times ([Adjusted odds ratio] aOR: 2.37, 95% CI: 1.77, 3.17) and children aged 19–23 months were 2.6 times (aOR: 2.6, 95% CI: 1.95–3.47) more likely to receive MAD. Children with early initiation of breastfeeding were 1.34 times (aOR: 1.34, 95% CI: 1.05, 1.72) and currently breastfeeding children were 4 times (aOR: 4.13, 95% CI: 2.21–7.69) more likely to receive MAD. Similarly, children with no sibling aged under 5 were 1.33 times (aOR: 1.33, 95% CI: 1.03, 1.73) more likely to receive MAD than those who had a sibling aged under 5 years. With an increment in one year of maternal age, a child was 3% (aOR: 0.97, 95% CI: 0.95–1.00) less likely to receive MAD. With an increment in one year of completed maternal education, there was a four percent (aOR: 1.04, 95% CI: 1.00–1.08) increase in the odds of having MAD among children. Children of mothers with media exposure had 1.66 (aOR: 1.66, 95% CI: 1.24, 2.21) times more odds of achieving MAD than their counterparts. Children from relatively disadvantaged ethnicity/caste were 29% (aOR: 0.71, 95% CI: 0.53, 0.94) less likely to receive MAD. Similarly, children of from rural areas were 1.45 times (aOR: 1.45, 95% CI: 1.06, 2.00) more likely to receive MAD than those from urban areas. In comparison to children from Bagmati Province, children from Madhesh province were 39% (aOR: 0.61, 95% CI: 0.37, 1.0) less likely to receive MAD (Table 4).

## 4. Discussion

To our knowledge, this is the first analysis conducted in Nepal on child feeding practices in accordance with the most recent World Health Organization (WHO) and UNICEF guideline for assessing infant and young child feeding practices, which was published in 2021 [6]. The prevalence of Minimum Dietary Diversity (MDD), Minimum Meal Frequency (MMF), Minimum Milk Feeding Frequency (MMFF) and Minimum Acceptable Diet (MAD) in this study were 39.8%, 68.5%, 17.8% and 30.1%, respectively. In comparison to data from the *Nepal Demographic and Health Survey 2016* (NDHS), the prevalence of MDD was lower by 7% and that of MAD was lower by 6% in this study. In contrast, the prevalence of MMF increased by about three percent compared to NDHS 2016. It is notable that the MDD and MAD were measured in NDHS 2016 with seven food groups, excluding breastmilk. Moreover, NDHS 2016 defined MDD as having received four or more of the seven food groups [8], whereas in our study, a child receiving five or more food groups was considered to have achieved MDD [6]. The prevalence of MDD was considerably higher in Nepal compared to other South Asian Countries (19%) [17], which had a MAD rate of 13% that is considerably lower than our present study [18].

By 2030, the government of Nepal aims to achieve the Sustainable Development Goals (SDGs) by reducing the prevalence of stunting to 15%, underweight to 9%, and the combined prevalence of wasting and overweight to 4% [19]. Every citizen of Nepal is guaranteed the right to food security and health under the country’s constitution. *National Health Policy 2019*, *National Nutrition Strategy 2021*, *Nepal Health Sector Strategy-III*, and *National Multi-Sector Nutrition Plan-II (2018–2022)* are now being implemented in Nepal to fulfill the constitutional requirement. In all 77 districts of Nepal, the Department of Health Services is implementing the IYCF program. The main nutritional interventions in Nepal also include iodine fortification in salt, integrated management of acute malnutrition program, iron and folic acid supplementation to pregnant and postpartum women, and biannual vitamin A supplementation and deworming campaigns [20]. Nepal has also joined Scaling-Up-Nutrition initiative, implementing several large-scale multisectoral and integrated projects for improving nutrition of children during the first 1000 days of life [4,21].

This study showed that the children aged 13–18 months and 19–23 months were more likely to receive MAD than children aged 6–12 months. This finding is consistent with previous studies conducted in Nepal [4,13,14,15] and countries in the South-Asian region [22]. A previous study showed that only 62% of children in Nepal are exclusively breastfed up to six months of age [5]. Though the “rice feeding ceremony” introduces boys and girls to complementary foods at six months and five months, respectively, in Nepal, children are not adequately and appropriately fed. Some communities believe that giving solid foods from animal sources to children under 12 months of age is inappropriate until their teeth have fully erupted [23]. So, concerns have been raised regarding the improvement of MDD, MMF and MMFF in children aged 6 to 12 months, as well as the timely introduction of complementary foods as other studies have noted [4,13].

This study showed that children who initiated breastfeeding within one hour of birth were more likely to receive MAD. One explanation could be that mothers of children who started breastfeeding early were more likely to give birth in health facilities and to have received information about newborn care and breastfeeding practices [24,25]. As expected, we found that children who were being breastfed had higher odds of having MAD. This is because the likelihood of achieving MDD and MMF, the components of MAD, is higher among currently breastfeeding children compared to the non-breastfeeding children.

Children without siblings aged under 5 years in the household were more likely to receive MAD because mothers can care for the single child better and offer them diverse foods more frequently without competition. A previous study found that a larger household size is a risk factor for food insecurity within households in Nepal [26]. Therefore, children with siblings under the age of five are more likely to receive food of inferior quality and quantity due to competing needs for food and health services.

Notably, we also found that a mother’s media exposure is associated with MAD among children. Studies from Nepal [12,15], other South-Asian countries [22], the East-African region [27] and Ethiopia [28] have also shown consistent positive association between a mother’s media exposure and the nutritional status of her children. Mother’s media exposure is associated with utilization of maternal health services like antenatal care, delivery in health institutions, postnatal care and family planning in similar settings [29,30,31]. In Nepal, information on maternal and child nutrition including IYCF is conventionally aired through mass media like television, radio and newspapers. For instance, the Suaahara II project transmits the *Bhanchin Aama* (Mother Says) radio show, public service advertisements about adolescent health and nutrition-related behaviors, and question-and-answer sessions called *Hello Bhanchin Aama* weekly in 40 project implementation districts [12,32]. A study among South-Asian countries showed that the effect of mass media is moderated by educational attainment of mothers [31]. It may be relevant to relate media exposure with a mother’s literacy in future. Furthermore, maternal education is an established determinant of positive IYCF behaviors [4,13,14,27]. Contrary to findings from earlier studies that older mothers were either more likely to achieve MAD [4,14,27] or maternal age is not associated with MAD [13], we found that older mothers were less likely to achieve MAD for their children.

We found that children from relatively underprivileged ethnic groups were less likely to receive MAD, and this is consistent with findings of Karn et al. from Nepal [4]. Additionally, among relatively underprivileged ethnicity/caste groups, the nutritional status of children, the use of child health care, and the use of maternal health services are all consistently low [33]. Such results may be attributable to lower educational attainment, poverty, and other socioeconomic problems among relatively disadvantaged ethnicity/caste groups. Similarly, children residing in Madhesh Province were less likely to have MAD than those living in Bagmati Province. This finding corroborates with the findings from another similar study conducted in Nepal [8]. Madhesh Province ranks second-to-last in terms of multidimensional poverty in Nepal [34]. Health-related measures like infant mortality, childhood malnutrition, and use of health services are all consistently low in the province [33]. These show that in addition to sociocultural influences, economic considerations and general development affect feeding behaviors, and these factors need to be addressed.

We found that children living in rural areas had greater rates of MAD than those living in urban areas. The finding, however, differs from that of the NDHS 2016, which showed that a comparable proportion of children in both rural and urban areas achieved MAD [8]. The higher prevalence of MAD in rural area might be attributed to the higher rate of continued breastfeeding of children up to one year among rural women in Nepal [5]. However, there is a potential for misclassification of rural and urban areas in this study as Nepal has recently reclassified some rural areas as urban areas despite no actual urbanization taking place [5].

Our study has several limitations. First, as the study used mother’s or caretaker’s responses to examine the events that occurred one or two years ago or the day before, recall bias cannot be avoided. Second, the 24-hour recall of the food intake of children might also be affected from social-desirability, and the frequency and diversity of the food intake may be overestimated. Third, given the cross-sectional nature of the study design, the results of this study cannot infer a causal relationship between MAD and other variables. Fourth, Multiple Indicator Cluster Surveys are not designed to assess factors relevant to IYCF practices, therefore some social, political, cultural and agricultural variables were not available for analysis. However, the study also has apparent strengths. We utilized nationally representative data to assess factors associated with MAD and used appropriate hierarchical analyses to address various design elements of the survey. Moreover, as this study is based on the latest definition of MAD by WHO and UNICEF, it can provide policymakers with up-to-date information to focus on specific groups to address the acute problem of child malnutrition in Nepal.

## 5. Conclusions

We found that age of the child, status of early initiation of breastfeeding, current breastfeeding status, maternal age, maternal education, ethnicity/caste, settlement and province of residence significantly predicted MAD among children. These findings can inform nutrition-specific programs and policies to improve IYCF practices and nutritional status among children aged 6 to 23 months in Nepal.

## Figures and Tables

**Table 1:** Description of explanatory variables.

Variables	Definition of Variables	Measurements
**Child’s characteristics**		
Age	Age of child in completed months	6–12 months, 13–18 months, 19–23 months
Sex	Sex of child	Boy, girl
Early initiation of breastfeeding	Child put to the breast within one hour of birth	Yes, no
Currently breastfeeding	Child breastfed on the previous day	Yes, no
Episode of diarrhea	Child had an episode of diarrhea within two weeks preceding the survey	Yes, no
Presence of sibling aged under five years	Child has a sibling aged under five years	Yes, no
**Mother’s characteristics**		
Age	Age of mother in completed years	15 to 49 years (Continuous numeric variable)
Education	Highest class attended	Grade 0–14 (0 indicates illiterate or no formal education 14 indicates Master’s degree or above) (Discrete numeric variable)
Media exposure	Mother listens to radio or reads newspaper or watches television at least once a week	Yes, no
Utilization of maternal health services	Mother had four antenatal visits and institutional delivery	Yes, no
**Household characteristics**		
Raw household wealth score	A composite score generated by principal component analysis using information on the ownership of consumer goods, dwelling characteristics, water and sanitation, and other assets and durables that are related to the household’s wealth	−1.89 to 2.42 (Continuous numeric variable)
**Ethnicity/Caste**	Ethnicity/caste of household members	Relatively advantaged: Brahmin, Chhetri, Newar, Gurung, Magar and Thakali, relatively disadvantaged: Others
**Area characteristics**		
Settlement	Level of urbanization	Urban (Metropolitan city, sub-metropolitan city, municipality), rural: Rural municipality
Province	Province where household is located	Province 1, Madhesh Province, Bagmati Province, Gandaki Province, Lumbini Province, Karnali Province, Sudoorpaschim Province

**Table 2:** Socio-demographic and healthcare-related characteristics of study participants.

Characteristics	Number (*n* = 1930)	Percentage (%)
**Age of child**		
6–12 months	642	36.4
13–18 months	625	30.5
19–23 months	663	33.1
**Sex of child**		
Girl	869	45.8
Boy	1061	54.2
**Early initiation of breastfeeding**		
No	1129	58.5
Yes	801	41.5
**Currently breastfeeding**		
No	105	6.4
Yes	1825	93.6
**Diarrhea in past two weeks**		
Yes	274	13.1
No	1656	86.9
**Has siblings aged under 5 years**		
Yes	739	38.0
No	1191	62.0
**Mother’s media exposure**		
No	740	37.3
Yes	1190	62.7
**Utilization of maternal health services**		
<4 ANC or no institutional delivery	623	33.7
4 ANC and institutional delivery	1307	66.3
**Ethnicity/caste**		
Relatively Advantaged	791	36.8
Relatively disadvantaged	1139	63.2
**Settlement**		
Rural	848	33.9
Urban	1082	66.1
**Province**		
Province 1	282	15.7
Madhesh Province	296	21.4
Bagmati Province	359	20.4
Gandaki Province	234	8.1
Lumbini Province	302	18.8
Karnali province	218	6.3
Sudoorpaschim Province	239	9.2

ANC: Antenatal Check-up.

**Table 3:** Association between minimum acceptable diet and child, maternal, household, and area factors among children aged 6–23 months in Nepal.

Characteristics	Minimum Acceptable Diet		Crude Odds Ratio
	No (*n* = 1346, %)	Yes (*n* = 578, %)	OR (95% CI)
**Age of child**			
6–12 months	551 (78.6)	150 (21.4)	Ref.
13–18 months	391 (66.5%)	197 (33.5%)	1.85 (1.34, 2.57) ***
19–23 months	405 (63.6%)	232 (36.4%)	2.1 (1.56, 2.83) ***
**Sex of child**			
Female	634 (71.9%)	248 (28.1%)	Ref.
Male	712 (68.3%)	330 (31.7%)	1.18 (0.95, 1.48)
**Early initiation of breastfeeding**			
No	789 (70.0%)	338 (30.0%)	Ref.
Yes	557 (69.9%)	241 (30.1%)	1.01 (0.79, 1.28)
**Currently breastfeeding**			
No	108 (87.0%)	16 (13.0%)	Ref.
Yes	1238 (68.8%)	562 (31.2%)	3.05 (1.03, 9.04) *
**Diarrhea in past two weeks**			
Yes	170 (67.2%)	83 (32.8%)	Ref.
No	1176 (70.4%)	495 (29.6%)	0.86 (0.63, 1.18)
**Has siblings aged under 5 years**			
Yes	564 (77.1%)	167 (22.9%)	Ref.
No	782 (65.6%)	411 (34.4%)	1.77(1.36, 2.30) ***
**Age of mother [Mean (SD)]**	25.93 (5.52)	25.51 (5.10)	0.99 (0.96, 1.01)
**Education of mother [Mean (SD)]**	6.62 (4.69)	8.26 (4.12)	1.09 (1.06, 1.12) ***
**Mother’s media exposure**			
Yes	567 (79.0%)	150 (21.0%)	Ref.
No	779 (64.5%)	428 (35.5%)	2.07 (1.62, 2.65) ***
**Utilization of maternal health services**			
<4 ANC or no institutional delivery	494 (76.1%)	155 (23.9%)	Ref.
4 ANC and institutional delivery	852 (66.8%)	423 (33.2%)	1.58 (1.19, 2.09) ***
**Combined household wealth score [Mean (SD)]**	0.04 (0.95)	0.23 (0.99)	1.23 (1.07, 1.41) ***
**Ethnicity/caste**			
Relatively advantaged	438 (61.8%)	270 (38.2%)	Ref.
Relatively disadvantaged	908 (74.7%)	308 (25.3%)	0.55 (0.42, 0.72) ***
**Settlement**			
Urban	898 (70.5%)	375 (29.5%)	Ref.
Rural	448 (68.8%)	203 (31.2%)	1.08 (0.84, 1.40)
**Province**			
Province 1	201 (66.4%)	102 (33.6%)	Ref.
Madhesh Province	323 (78.3%)	89 (21.7%)	0.55 (0.35, 0.85) **
Bagmati Province	243 (61.7%)	151 (38.3%)	1.23 (0.82, 1.84)
Gandaki Province	94 (60.2%)	62 (39.8%)	1.31(0.86, 1.98)
Lumbini Province	266 (73.4%)	96 (26.6%)	0.71(0.46, 1.10)
Karnali Province	94 (77.6%)	27 (22.4%)	0.57 (0.34, 0.96) *
Sudoorpaschim Province	126 (71.3%)	51 (28.7%)	0.79 (0.52, 1.22)

* p < 0.05 ** *p* < 0.01 *** *p* < 0.001, OR: Odds Ratio, CI: Confidence Interval, Ref.: Reference, ANC: Antenatal Check-up, SD: Standard Deviation.

**Table 4:** Multilevel analysis for predictors of minimum acceptable diet among children aged 6–23 months in Nepal.

Predictors	Model 0	Model 1		Model 2		Model 3	
		aOR	95% CI	aOR	95% CI	aOR	95% CI
Age: 13–18 months (Ref: 6–12 months)		2.15 ***	1.61, 2.85	2.36 ***	1.76, 3.15	2.37 ***	1.77, 3.17
Age: 19–23 months (Ref: 6–12 months)		2.34 ***	1.77, 3.10	2.60 ***	1.95, 3.46	2.60 ***	1.95, 3.47
Sex of children: Male (Ref: Female)		1.17	0.93, 1.47	1.14	0.90, 1.43	1.15	0.91, 1.45
Early initiation of breastfeeding: Yes (Ref: No)		1.21	0.95, 1.53	1.28 *	1.01, 1.63	1.34 *	1.05, 1.72
Currently breastfeeding: Yes (Ref: No)		3.94 ***	2.12, 7.29	4.26 ***	2.28, 7.93	4.13 ***	2.21, 7.69
Diarrhea in past two weeks: No (Ref: Yes)		0.83	0.59, 1.16	0.79	0.56, 1.12	0.79	0.56, 1.11
Having siblings aged U5: No (Ref: Yes)		1.72 ***	1.34, 2.22	1.42 **	1.10, 1.84	1.33 *	1.03, 1.73
Age of mother				0.98 *	0.95, 1.00	0.97 *	0.95, 1.00
Mother’s education				1.04 *	1.01, 1.08	1.04 *	1.00, 1.08
Media exposure: Yes (Ref: No)				1.71 ***	1.29, 2.28	1.66 ***	1.24, 2.21
Health service utilization: 4 ANC and institutional delivery (Ref: Otherwise)				1.15	0.86, 1.54	1.14	0.85, 1.53
Combined household wealth score				1.07	0.91, 1.25	1.09	0.90, 1.32
Ethnicity/caste: Relatively disadvantaged (Ref: Relatively advantaged)				0.70 **	0.53, 0.91	0.71 *	0.53, 0.94
Settlement: Rural (Ref: Urban)						1.45 *	1.06, 2.00
Province: Province 1 (Ref: Bagmati Province)						0.99	0.61, 1.59
Province: Madhesh Province (Ref: Bagmati)						0.61 *	0.37, 1.00
Province: Gandaki Province (Ref: Bagmati)						1.00	0.59, 1.69
Province: Lumbini Province (Ref: Bagmati)						0.66	0.41, 1.05
Province: Karnali Province (Ref: Bagmati)						0.53	0.26, 1.05
Province: Sudoorpaschim Province (Ref: Bagmati)						0.62	0.35, 1.11
**Random Effects**							
Intraclass correlation (%)	20%		18%		17%		17%
Marginal R^2^/Conditional R^2^	0.000/0.196		0.133/0.286		0.140/0.289		0.159/0.300
**AIC**	2291		2164		2161		2159
**BIC**	2303		2236		2244		2281
**−2 Log Likelihood**	−1144 (Ref.)		−1069		−1065		−1057
**Deviance**	2287		2138		2131		2115

* *p* < 0.05 ** *p* < 0.01 *** *p* < 0.001. Model 0: Null Model; Model 1: Individual Model; Model 2: Individual, Maternal and Household Model; Model 3: Individual, Maternal, Household and Area Model; aOR: Adjusted Odds ratio; CI: Confidence Interval; ANC: Antenatal check-up; AIC: Akaike Information Criterion; BIC: Bayesian Information Criterion.

## Data Availability

The datasets used for analyses in this study are publicly available upon request from MICS website: https://mics.unicef.org/surveys, accessed on 5 January 2022.
